# Correction of Diabetic Hyperglycemia and Amelioration of Metabolic Anomalies by Minicircle DNA Mediated Glucose-Dependent Hepatic Insulin Production

**DOI:** 10.1371/journal.pone.0067515

**Published:** 2013-06-27

**Authors:** Tausif Alam, Philip Wai, Dustie Held, Sahar Taba Taba Vakili, Erik Forsberg, Hans Sollinger

**Affiliations:** Division of Transplantation, Department of Surgery, University of Wisconsin, Madison, Wisconsin, United States of America; National Institutes of Health, United States of America

## Abstract

Type 1 diabetes mellitus (T1DM) is caused by immune destruction of insulin-producing pancreatic β-cells. Commonly used insulin injection therapy does not provide a dynamic blood glucose control to prevent long-term systemic T1DM-associated damages. Donor shortage and the limited long-term success of islet transplants have stimulated the development of novel therapies for T1DM. Gene therapy-based glucose-regulated hepatic insulin production is a promising strategy to treat T1DM. We have developed gene constructs which cause glucose-concentration–dependent human insulin production in liver cells. A novel set of human insulin expression constructs containing a combination of elements to improve gene transcription, mRNA processing, and translation efficiency were generated as minicircle DNA preparations that lack bacterial and viral DNA. Hepatocytes transduced with the new constructs, *ex vivo*, produced large amounts of glucose-inducible human insulin. *In vivo,* insulin minicircle DNA (TA1m) treated streptozotocin (STZ)-diabetic rats demonstrated euglycemia when fasted or fed, *ad libitum*. Weight loss due to uncontrolled hyperglycemia was reversed in insulin gene treated diabetic rats to normal rate of weight gain, lasting ∼1 month. Intraperitoneal glucose tolerance test (IPGT) demonstrated *in vivo* glucose-responsive changes in insulin levels to correct hyperglycemia within 45 minutes. A single TA1m treatment raised serum albumin levels in diabetic rats to normal and significantly reduced hypertriglyceridemia and hypercholesterolemia. Elevated serum levels of aspartate transaminase, alanine aminotransferase, and alkaline phosphatase were restored to normal or greatly reduced in treated rats, indicating normalization of liver function. Non-viral insulin minicircle DNA-based TA1m mediated glucose-dependent insulin production in liver may represent a safe and promising approach to treat T1DM.

## Introduction

Type 1 diabetes mellitus (T1DM) results from the autoimmune destruction of insulin-producing β cells of the pancreas. There is no cure for T1DM, only insulin therapy. Injectable insulin preserves life but perfect glucose control is difficult to achieve and chronic hyperglycemia-associated systemic damage takes its toll. Diabetes is the leading cause of end-stage kidney failure, blindness, and non-traumatic amputations. In the US, approximately 1–1.5M patients, most of them children, have been diagnosed with T1DM. The economic cost in the US is estimated at $8–$14B/year. Transplantation of pancreas or pancreatic islets achieves excellent glucose control in T1DM patients but its usefulness is limited due to a severe shortage of donor organs. A method to control blood glucose without multiple daily insulin injections that (1) is suitable for all patients with T1DM, (2) requires no major operative procedure or intervention, (3) is affordable for all patients, and (4) requires no or only minimal immunosuppressive therapy is an urgent, unmet need.

Many alternative approaches to treat T1DM with cells engineered to produce insulin, without using donor pancreata, have been attempted. Examples of such attempts include insulin production from immortalized cells [1], [2], 3,4 requiring containment to avoid dangerous expansion; various native cells, such as liver cells [5], [6], [7], [8], [9], [10], gastric G cells [11], [12], K cells [13], [14], L cells [15], muscle cells [16], [17], [18], fibroblasts [19], [20], [21]; and recently, recipient’s non-β-cells reprogrammed to function as β-cells. The last category includes direct reprogramming of terminally differentiated developmentally closely related cells such as the liver and exocrine pancreas [22], [23], [24], [25], [26], [27], and use of embryonic stem cells as well as induced pluripotent cells to produce β-cells [20], [28], [29], [30], [31]. Most approaches have significant limitations either due to concerns of safety in their use, their limited functional efficacy, or both. Insulin production in many of these studies was inadequate to fully correct postprandial hyperglycemia in diabetic animals. Subnormal glucose disposal rate was apparent in several studies as exhibited by abnormal glucose tolerance test profiles even when a relatively low glucose load (only 0.5–1.5gm/kg) was used [10], [25], [27], [32]. In many early approaches, glucose-independent promoters directed insulin synthesis followed by its constitutive secretion which does not provide adequate control of blood glucose [8], [18]. Additionally, commonly employed vectors contain DNA from bacterial or viral origins which add to the safety concerns and reduce the longevity of gene expression [33].

Experimental approaches to treat T1DM by the *in vivo* reprogramming of recipient’s native cells directed by expression of transcription factors, such as PDX1 [22], [23], [27], [34], Neurogenin 3, and Neuro D [25], [26] have met some success but many long-term safety concerns remain to be addressed. Additionally, considerable efforts are being made to use human embryonic stem (hES) cells and induced pluripotent stem (iPS) cells as renewable sources to generate large quantities of β-cells [31], [35], [36]. However, use of hES and iPS cell derived β-cells to treat T1DM is hampered by significant hurdles; the limited yield, questionable purity, possible tumorigenecity and the functional maturity of neo-β-cells.

In this study we report novel insulin constructs in minicircle DNA that drive glucose-dependent insulin production in livers of streptozotocin (STZ)-induced diabetic rats. The insulin output adequately corrects diabetic hyperglycemia and restores many diabetes-associated parameters of anomalous metabolism to normal, including the normal rate of weight gain in insulin gene-treated diabetic animals.

## Materials and Methods

### Insulin Gene Construct Preparation

Five insulin constructs were generated by combining the native human insulin coding sequence, modified at B-C (KTRR–>RTKR) and C-A (Leu –>Arg) junctions of proinsulin to render it furin cleavable, albumin promoter, and 3 tandem repeats of glucose inducible regulatory elements (GIREs) (described previously [6]), with human α-fetoprotein enhancer, human growth hormone intron, human VEGF derived transcriptional enhancer, and human albumin 3′-untranslated region, in the order indicated in Figure 1A. Polymerase chain reaction and other standard techniques of molecular biology and cloning were used to prepare these insulin gene constructs. Sequence analysis of all five constructs, termed TA0, TA1, TA2, TA3, and TA4 (Figure 1A), confirmed the integrity of the constructs.

**Figure 1:**
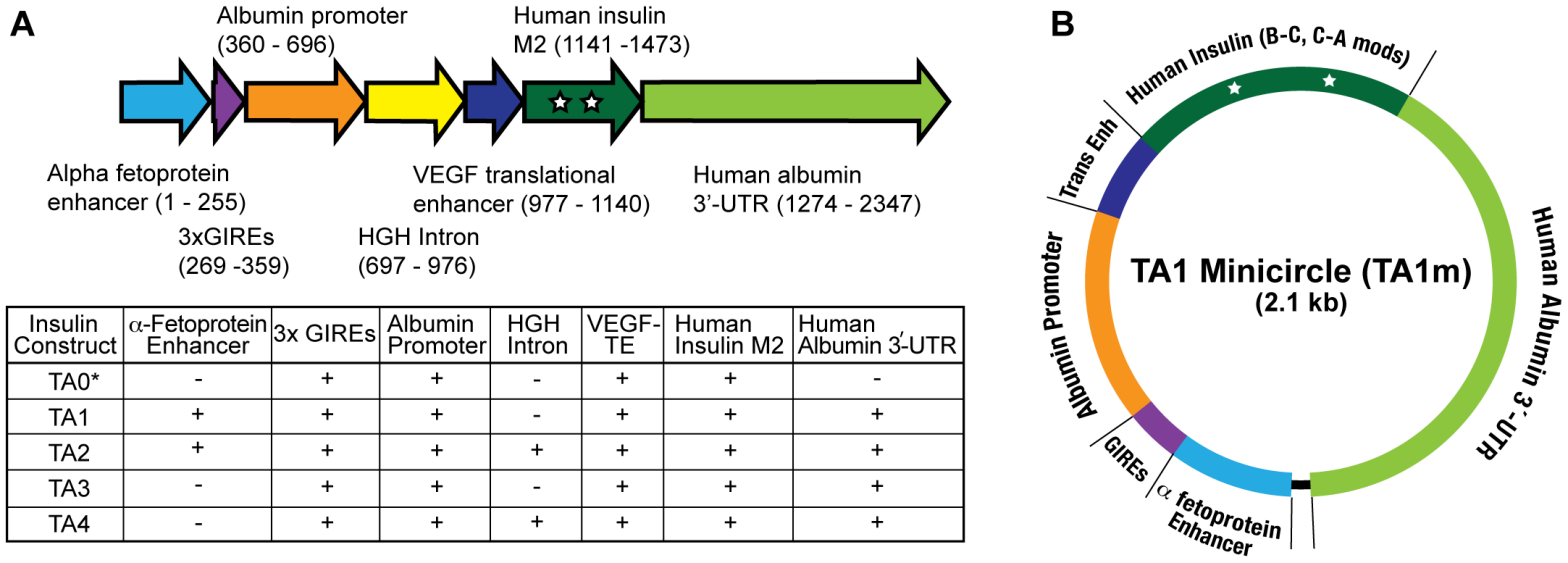
Insulin gene constructs. A: All 5 human insulin gene expression constructs used in this study contained a core of 3x GIREs, albumin promoter, VEGF derived translation enhancer, and B–C and C–A junction modified human preproinsulin coding sequence. The TA1– TA4 also contained human albumin 3′-UTR and a combination of α-fetoprotein enhancer and HGH-intron, as indicated above. B: For illustrative purpose, the minicircle TA1m is shown to highlight the absence of bacterial or viral DNA sequences that are common in all other vectors and implicated in gene destruction or silencing through multiple mechanisms.

All insulin gene constructs were cloned into a replication-defective adenovirus using pAd/PL-DEST Gateway Vector from Life Technologies (NY, USA). The five insulin constructs in adenovirus were designated as Ad-TA0, Ad-TA1–Ad-TA4 and used for *ex vivo* insulin expression in primary hepatocytes and for transient hepatic expression of insulin *in vivo* for the purpose of evaluation. All adenovirus preparations were purified by cesium chloride equilibrium density gradient and the titers determined by a functional assay, by measuring the ability of adenovirus to infect HEK 293 cells and produce hexon protein, which was detected by immunocytochemistry using a reagent kit from Cell Biolabs, Inc. (San Diego, CA). Four insulin expression constructs (TA1–TA4) were also cloned into the parental plasmid from System Biosciences (Mountain View, CA) [37] after removing all native sequences between attB and attP recombination sites. The minicircle DNA preparations corresponding to TA1–TA4 were produced in E. coli ZYCY10P3S2T using the procedure provided by the supplier, and termed TA1m, TA2m, TA3m, and TA4m, respectively. The minicircle DNA preparations were purified using EndoFree reagents from Qiagen (Valencia, CA). A representative drawing of TA1m is shown in Fig. 1B for illustration.

### *In Vitro* Insulin Production from Transduced Hepatocytes

Hepatocytes were prepared from normal Wistar rat livers by in situ perfusion of collagenase (≥90% viability, as judged by eosin exclusion), as described previously [6]. Hepatocytes were plated on collagen-coated culture plates in Roswell Park Memorial Institute 1640 culture medium containing 10% fetal bovine serum, 5 µg/mL transferrin, 5 µg/mL selenium, and 15mM glucose. After adhesion of hepatocytes (3–4 hr), the medium was replaced with fresh medium containing 3.3 mM glucose and adenovirus (MOI = 50) containing one of the 5 insulin constructs (AdTA0, AdTA1, AdTA2, AdTA3, or AdTA4), as indicated. The controls included cells transfected with β-galactosidase containing adenovirus, AdCMV.β-Gal [6], and untransfected normal hepatocytes. The culture medium was replaced 16 hr after transfection with fresh medium containing indicated concentrations of glucose. Secreted insulin in the culture medium was measured by enzyme-linked immunosorbent assay (ELISA) [6]. Each test was performed in triplicate, and all of the experiments have been repeated at least three times.

### *In vivo* Insulin Gene Therapy of Streptozotocin-Induced Diabetic Rats

Normal male Wistar rats, weighing 60- to 200g, were temporarily restrained and injected with streptozotocin (STZ) either into their peritoneum or tail veins at a dose of 100mg/kg. The STZ treatment rendered >90% rats diabetic in within 24 h. The STZ-diabetic rats with fasting blood glucose >350mg/dl were divided into indicated experimental groups. Rats to be treated with adenovirus were anesthetized using isoflurane and a 2- to 3cm midline incision made to gain access to their livers. The indicated amounts of adenovirus were injected directly into the liver at multiple sites in 0.5 mL of saline and the incisions were closed. The control diabetic rats were similarly injected with either a comparable amount of unrelated adenovirus containing bacterial β-galactosidase, AdCMV.β-Gal, or saline. Rats to be treated with minicircle DNA were temporarily restrained and the specified minicircle DNA in TransIT EE Hydrodynamic Delivery Solution (Mirus Bio, Madison, WI) in a 10% volume, relative to body weight, was injected through the tail vein [38]. Periodic fasting and *ad libitum* fed blood glucose levels, and body weight of all the rats were measured. Tissue samples used for detection of insulin mRNA from various groups of experimental rats were excised 4 days after adenovirus treatment, frozen in liquid nitrogen, and stored at −70°C until used for RNA extraction.

All animal studies were conducted under animal protocols approved by the School of Medicine and Public Health Animal Care and Use Committee (IACUC) of University of Wisconsin at Madison. Rats used for isolation of hepatocytes were anesthetized by inhalation of isoflurane. *In situ* infusion of collagenase and exsanguination, which is a part of the hepatocyte preparation protocol, caused death of rats under anesthesia. Experimental animals were not subjected to any procedures that may cause prolonged pain and were humanely euthanized under anesthesia at the end of the experiments during the process of final blood and tissue sample collection. In accordance with PHS policy on Humane Care and Use of Laboratory Animals of the NIH, rats becoming sick or accidentally injured were euthanized by hypoxia using carbon dioxide. Rodents were placed in a non-precharged chamber and 100% CO_2_ was introduced at a rate of 10–20% of the chamber volume per minute; rats were kept in the chamber until clinically dead.

### Detection of Insulin in sections of Liver and Pancreas by Immunocytochemistry

Liver, pancreas, spleen, kidney, and muscle tissues from normal, STZ-diabetic, and STZ-diabetic rats treated with various insulin constructs were excised 4 days after adenovirus or minicircle treatment, fixed in phosphate buffered 10% formalin (containing 3.7%–4% formaldehyde) solution overnight, and processed [39]. Immunocytochemical detection of insulin in 5-µm-thick tissue sections was performed by using the guinea pig anti-porcine insulin primary antibody, which cross-reacts with human insulin, and peroxidase conjugated secondary antibody, rabbit anti–guinea pig immunoglobulin IgG (Sigma Chemical Co., St. Louis, MO). Chromogenic ImmPACT DAB peroxidase substrate was purchased from Vector Laboratories, Inc., Burlingame, CA and used according to the supplied procedure. Insulin stained liver sections were lightly stained with hemotoxylin to visualize nuclei. All tissue sections from each animal were also stained with hemotoxylin and eosin and analyzed for T-cell infiltration.

### Quantitation of Human Insulin in Rat Plasma by ELISA

All measurements of plasma levels of human insulin in rats treated with insulin gene constructs were performed using commercial “Ultrasensitive Insulin ELISA” reagents from Mercodia (Mercodia Inc, Winston Salem, NC, USA). According to the data provided by the supplier, this method is human insulin specific and rat and mouse insulin cross-reactivity is well below 1%. This ELISA also does not cross-react with C-peptide or unprocessed proinsulin (<0.01%), proinsulin des (31–32; <0.5%), proinsulin des (32–33; <0.5%). However, proinsulin des (64–65) and proinsulin des (65–66) show significant cross-reactivity at 98% and 56%, respectively.

## Results

### Insulin Gene Constructs

All insulin gene constructs used in this study (Figure 1A) include 3 tandem repeats of GIREs derived from transcription regulator S14 and albumin promoter upstream from the human pre-proinsulin cDNA modified at B-C and C-A junctions to facilitate proteolytic maturation of insulin by furin in liver cells. This configuration of elements ensures hepatocyte specific glucose-dependent insulin expression, as described previously [6]. Four insulin gene constructs, TA1, TA2, TA3, and TA4, included additional components (Fig. 1A) to collectively improve the efficiency of the constructs in producing much larger amount of insulin than previously described construct 3SAM2. These additional components include a transcriptional enhancer from human α-fetoprotein sequence, a segment of human growth hormone DNA containing an intron to improve RNA processing efficiency, a translation enhancer from human VEGF sequence that functions as an internal ribosomal entry site, and DNA containing the 3′-untranslated region of human albumin sequence that includes an intron to facilitate mRNA processing. The construct TA0 contained only the VEGF translational enhancer in addition to GIREs, albumin promoter, and the modified human insulin sequence; it lacked α-fetoprotein enhancer, HGH intron, and albumin 3′-UTR.

The relative ability of each of the 5 insulin gene constructs (TA0, TA1-TA4) to produce glucose-dependent insulin was initially compared by transducing primary rat hepatocytes *ex vivo* with adenovirus vector containing insulin (Ad-TA0, AdTA1–Ad-TA4); results are shown in Fig. 2. All insulin constructs caused glucose-induced insulin production. Insulin constructs TA1–TA4 caused significantly larger amount of insulin production (6–14 times higher) than TA0 that lacked α-fetoprotein enhancer, HGH intron, and human albumin 3′-UTR. It is worthwhile to note that incorporation of VEGF derived translational enhancer alone in the previously published construct Ad3SAM2 [6] caused a 4–6 fold increase in insulin output at low and high glucose concentrations from transduced hepatocytes under comparable conditions (data not shown). Insulin constructs TA1 and TA4 performed significantly better than TA3 in *ex vivo* tests (Fig. 2) which lacked α-fetoprotein enhancer and HGH intron. In the ex vivo testing, the Ad-TA2, containing α-fetoprotein enhancer and HGH intron, produced less insulin than constructs containing either one of the two elements.

**Figure 2:**
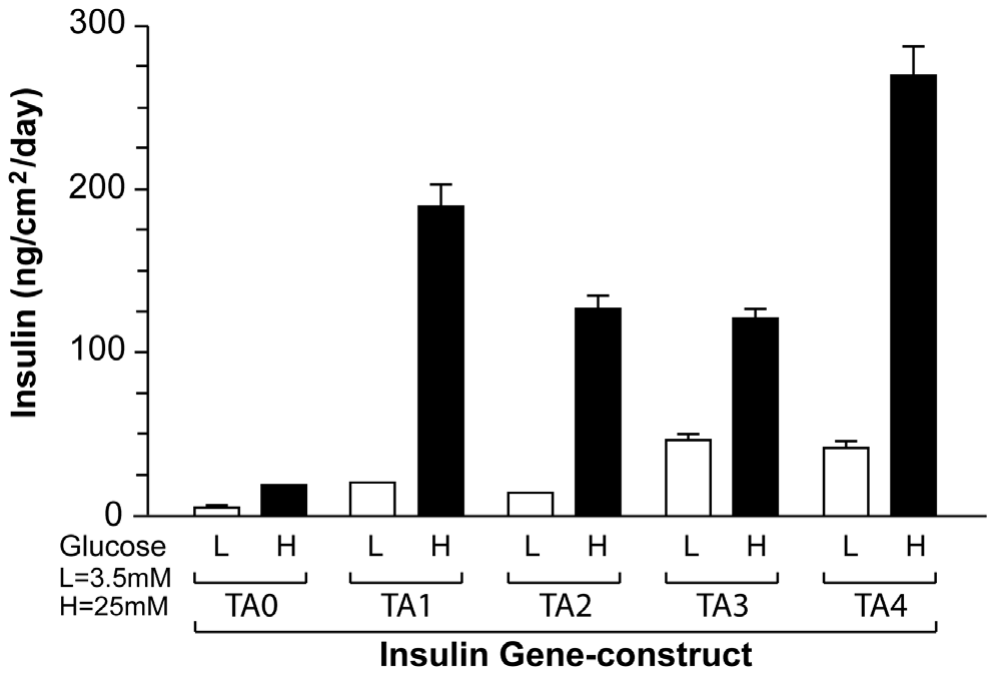
Glucose-dependent ex vivo insulin production from adenovirus transduced hepatocytes. Freshly isolated rat hepatocytes were plated in collagen coated 6-well cell culture plates, as described in methods. Equal number of hepatocytes were used in each well (1.2×10^6^ hepatocytes/well). Hepatocytes were transduced in triplicate with each of the indicated insulin gene construct in adenovirus at the constant multiplicity of infection (MOI = 50), as described in methods. Two sets of control wells were used; one set included a matched number of adenovirus containing bacterial β-galactosidase (Ad-CMV.β-Gal) and the other an equal volume of carrier solution (saline). An aliquot of ∼100 µl culture medium was removed at 12 h, 24 h, 36 h, 48 h, and 72 h time points after the initial exposure to high (25mM) or low glucose (2.5mM). Insulin produced and secreted by the transduced hepatocytes was assayed; at least duplicate measurements were made at multiple dilutions. Both sets of control wells showed no detectable insulin. Peak insulin was produced at 36 h–48 h time points. For ease of comparison, the insulin amount is shown in ng/day in a unit surface area of the cell culture plate. These experiments have been repeated 3 times with similar results.

### Treatment of STZ-diabetic Rats with Insulin Gene Constructs

The insulin constructs TA1 and TA4 in adenovirus were tested for their ability to correct hyperglycemia in STZ-treated diabetic rats. The 15-day (d7–d21) average (mean±SD) blood glucose levels of overnight fasting diabetic rats treated with either Ad-TA1 (101±18mg/dl) or Ad-TA4 (118±9mg/dl) were significantly reduced when compared to the diabetic rats (450±49mg/dl, P<0.0001). The Ad-TA1 treated STZ-rats had a normal fasting glucose level with no statistically significant difference between them and the normal controls (87±11mg/dl) (Fig. 3A). The construct Ad-TA1 performed slightly better than the Ad-TA4 as evidenced by a significant difference in fasting blood glucose levels of normal and Ad-TA4 treated STZ-rats. Although the glucose levels in adenovirus treated rats, fed *ad libitum*, were not fully corrected, they were significantly lowered (d8–d24 mean±SD, Ad-TA1 and Ad-TA4 treated rats: 363±25mg/dl, 353±43mg/dl, respectively) when compared to the STZ-diabetic rats 625±75mg/dl (P<0.0001) (Fig. 3B). The difference in ad libitum fed Ad-TA1 and Ad-TA4 treated diabetic and normal rats (131±7mg/dl) was also significant (P<0.0001), but there was no significant difference between the glucose levels of Ad-TA1 and Ad-TA4 treated rats (Fig. 3B). The effect of a single insulin gene treatment using either Ad-TA1 or Ad-TA4 on restoration of physical growth of diabetic rats was striking. While the untreated diabetic rats stopped gaining weight or gradually lost weight after STZ treatment, the Ad-TA1 or Ad-TA4 treated rats appeared healthy and gained weight at or nearly the same rate as the normal controls (Fig. 4). The group of STZ-diabetic rats treated with Ad-TA1 and Ad-TA4 gained weight at a rate of 3.1±1.3gm/rat/day and 3.2±1.3gm/rat/day during the first 20 days after the treatment, which is statistically indistinguishable from the rate of weight gain for age-matched normal rats (3.2±0.5gm/rat/day) during the same period. The uncontrolled diabetic rats lost significant weight soon after STZ treatment and their growth was severely retarded (−0.01±0.81gm/rat/day) (Fig. 4).

**Figure 3:**
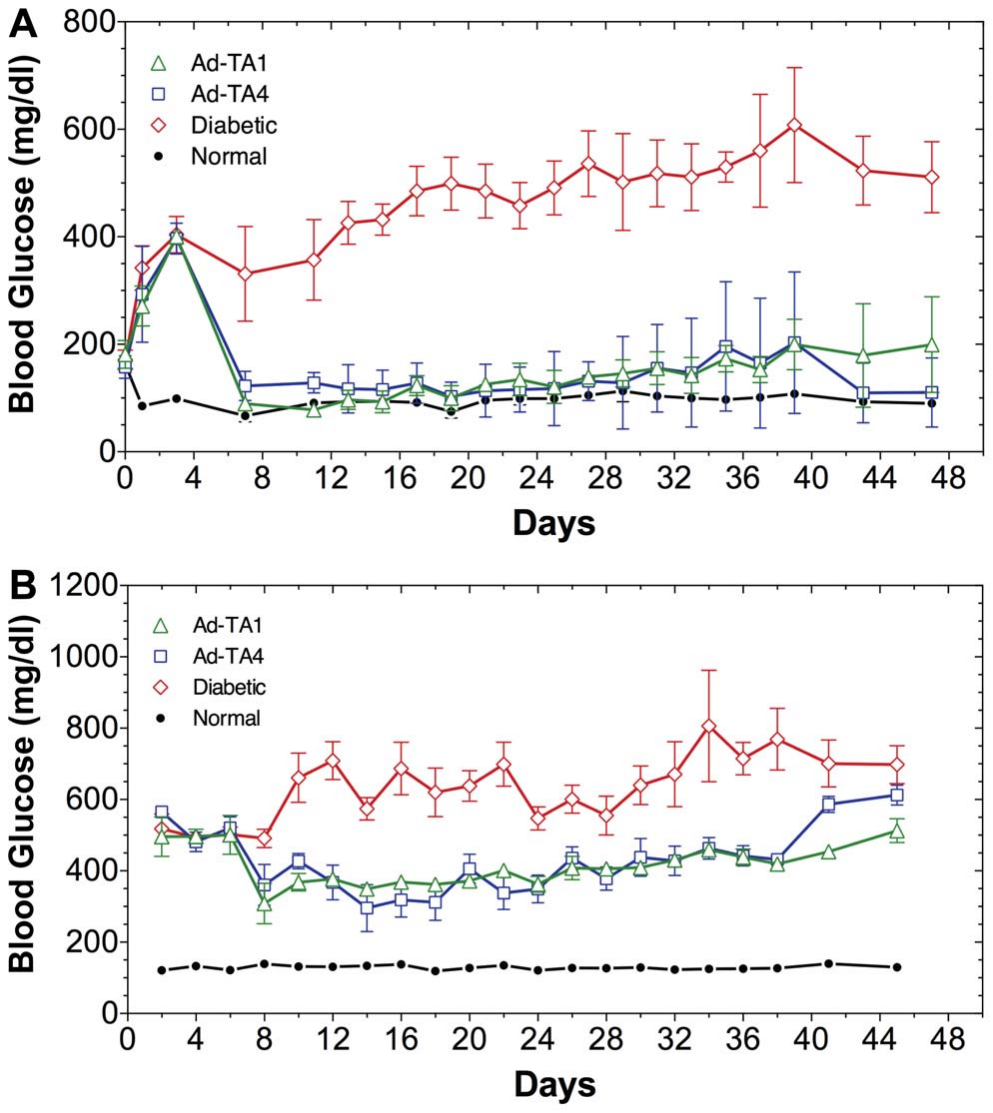
Effects of in vivo hepatic insulin production on hyperglycemia of diabetic rats. Five groups of age matched rats (6 rats/group) weighing ∼175g were intravenously treated with 100mg/kg STZ to render them diabetic. On day 3 after STZ treatment, rat livers were injected with 2×10^10^ adenovirus vector pfu/rat, as described in methods. Each group of diabetic rats was treated with equal adenovirus vector particles (Ad-TA1 or Ad-TA4), as indicated in the figure. One group of control diabetic rats was injected with 2×10^10^ pfu/rat Ad-CMV.β-Gal and the other with equal volume of saline. Both groups of diabetic control rats yielded similar results. A group of healthy rats was also included as normal controls. Blood glucose levels and body weights of each rat were recorded daily; the measurements were alternated between rats fasted overnight and fed *ad libitum*. These experiments have been repeated at the minimum 3 times. A: 16 h fasting blood glucose levels from: open green triangle: Ad-TA1 treated STZ-rats; open blue square: Ad-TA4 treated STZ-rats; open red diamond: untreated STZ-diabetic rats; closed black circles: normal control rats. B: Alternate day *ad libitum* fed measurements of blood glucose levels from experimental animals described in Figure 3A.

**Figure 4:**
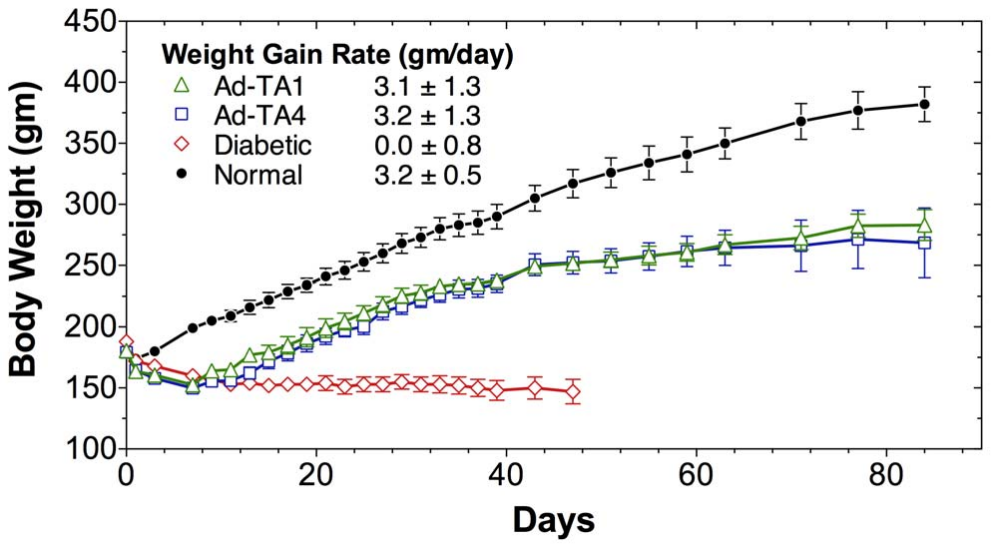
Effects of in vivo hepatic insulin production on body weights of diabetic rats. Body weights of all experimental groups of rats described in Fig. 3 were measured after a 16 h fast. Symbols for various groups of experimental rats are the same as in Fig. 3; open green triangle: Ad-TA1 treated STZ-rats; open blue square: Ad-TA4 treated STZ-rats; open red diamond: untreated STZ-diabetic rats; closed black circles: normal control rats. Rate of weight gain (gm/day) in each group of rats was calculated and shown as mean±standard deviation.

### Treatment of STZ-diabetic Rats with Insulin Gene Constructs in Minicircle DNA

We tested TA1m at DNA doses ranging from 0.25–1.0 µg/gm body weight, as indicated (Fig. 5), for its ability to correct hyperglycemia in STZ-diabetic rats. We observed a DNA dose-dependent correction in hyperglycemia of STZ-rats, fed *ad libitum* (Fig. 5A), and fasted (Fig. 5B). A full restoration of rate of weight gain in young diabetic rats (initial body-weight = 46±2gm), comparable to normal young rats, was noted in all groups that were treated with TA1m at 0.5–1.0 µg/gm body-weight (Fig. 6). Only the diabetic rats treated with the lowest DNA dose (0.25 µg/gm body-weight) were unable to attain the growth rate of normal controls (Fig. 6). The difference between the effectiveness of TA1m and TA2m treatment of diabetic rats, as judged by the rate of weight gain (Fig. 7), was insignificant. However, TA3m, lacking α-fetoprotein enhancer and HGH intron, was far less effective in correcting diabetic hyperglycemia and normalizing the rate of weight gain (Fig. 7). Use of minicircle DNA holds the promise of repeated effective treatments. Our preliminary results shown in the Fig. 8 support the notion that when the effect of the first treatment begins to diminish, a second treatment is effective in reducing hyperglycemia.

**Figure 5:**
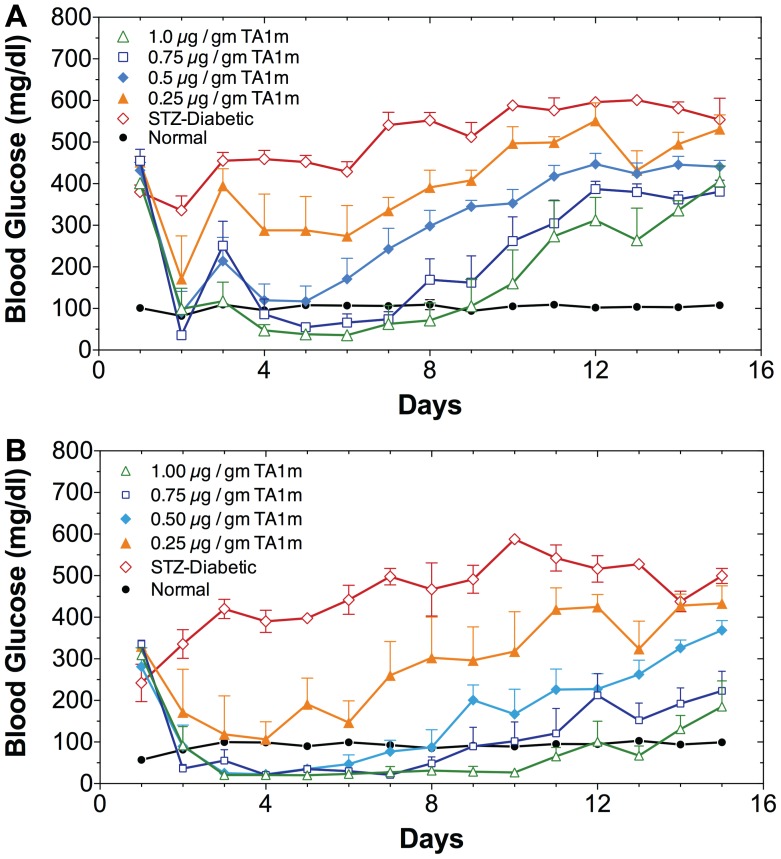
DNA dose-dependent effect of insulin minicircle treatment on hyperglycemia of young diabetic rats. Five groups of age matched rats (5–7 rats/group) weighing ∼50gm were treated intraperitoneally with 100mg/kg STZ to render them diabetic. Two days after STZ treatment, rats were intravenously injected with indicated dose of TA1m at high pressure. In this experiment, four TA1m doses, 0.25 µg/gm body weight, 0.50 µg/gm body weight, 0.75 µg/gm body weight, and 1.0 µg/gm body weight, were used. A group of untreated diabetic rats and a group of normal rats were included as controls. Blood glucose levels and body weights of each rat were recorded daily; the measurements were alternated between rats fasted and fed *ad libitum*. These experiments have been repeated at the minimum 3 times. A: All blood glucose level measurements are made on rats fed *ad libitum*. Orange filled triangle: 0.25 µg/gm TA1m treated STZ-rats; blue filled diamond: 0.50 µg/gm TA1m treated STZ-rats; open black square: 0.75 µg/gm TA1m treated STZ-rats; open green triangle: 1.00 µg/gm TA1m treated STZ-rats; open red diamond: untreated STZ-rats; filled black circle: normal control rats. B: Alternate day measurements of blood glucose levels after a 4 h fast from experimental animals are shown, as indicated.

**Figure 6:**
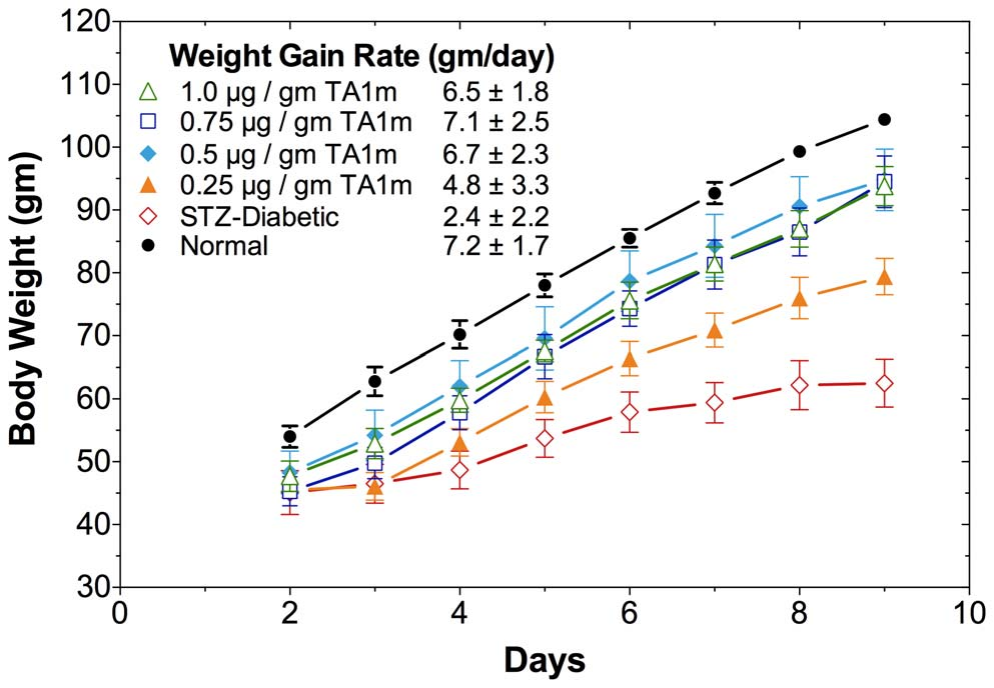
DNA dose effect of insulin minicircle treatment on growth of young diabetic rats. Body weights of all experimental groups of rats described in Fig. 5 were measured after a 4 h fast. The TA1m doses and symbols for various groups of experimental rats are the same as shown in Fig. 5. Orange filled triangle: 0.25 µg/gm TA1m treated STZ-rats; blue filled diamond: 0.50 µg/gm TA1m treated STZ-rats; open black square: 0.75 µg/gm TA1m treated STZ-rats; open green triangle: 1.00 µg/gm TA1m treated STZ-rats; open red diamond: untreated STZ-rats; filled black circle: normal control rats. Rate of weight gain (gm/day) in each group of rats was calculated and shown as mean±standard deviation.

**Figure 7:**
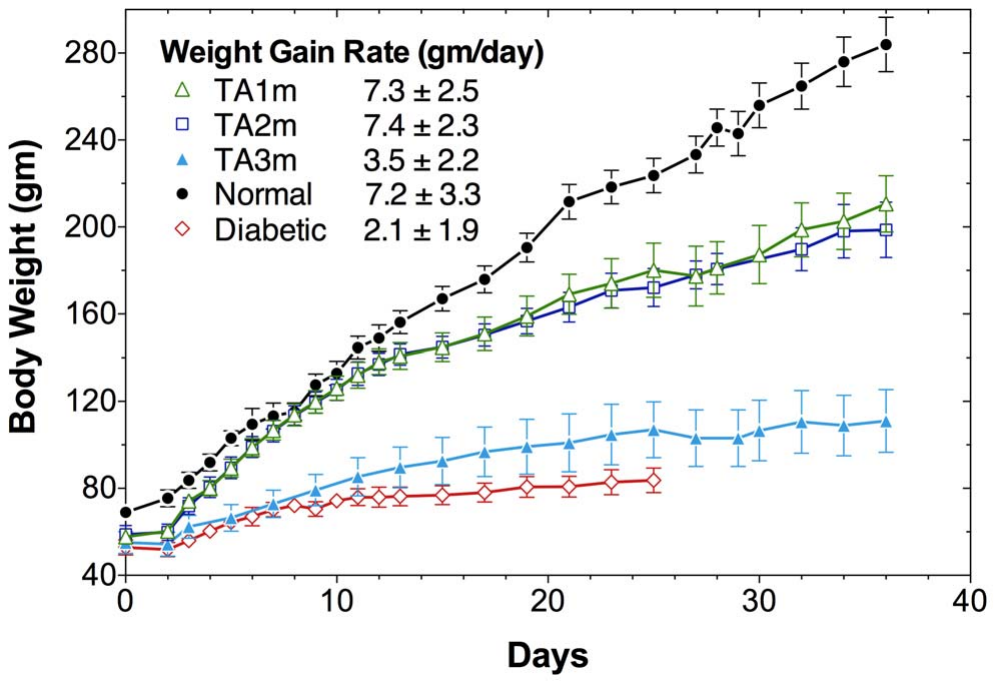
Comparison of TA1m, TA2m, and TA3m insulin minicircle DNA constructs in treating diabetes. Groups of young diabetic rats were treated with minicircle DNA via tail vein high-pressure injections, as described in Figure 5 and methods. A: Body weights of *ad libitum fed* diabetic rats treated with TA1m at 0.8 µg/gm body-weight (green open triangles); with TA2m (blue open square); and with TA3m (blue filled triangle). The DNA dose used for TA2m and TA3m were equimolar to the TA1m dose. Red open diamond: body weights of ad libitum fed untreated diabetic rats; black filled circles: body weights of ad libitum fed normal control rats. Rate of weight gain/day, shown in the inset, represents mean±standard deviation for each group of rats.

**Figure 8:**
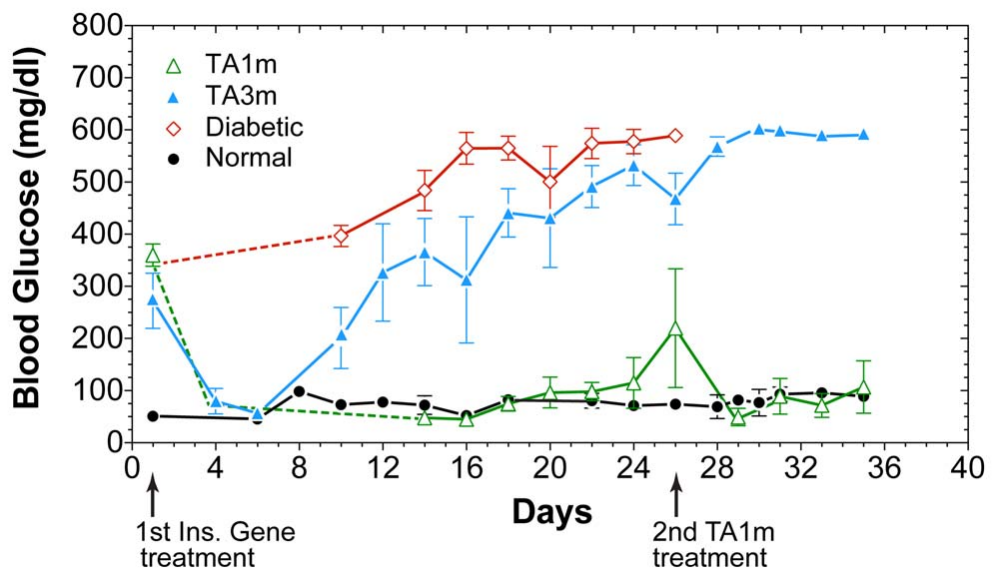
Effect of second TA1m treatment on fasting hyperglycemia of diabetic rats. Groups of young STZ-diabetic rats were treated with TA1m DNA at 0.8 µg/gm body-weight or molar equivalent of TA3m via tail vein high-pressure injections on day 1, as described in methods. Rats were fasted for 4 h and blood glucose levels of all groups of rats were measured as indicated. Green open triangles: TA1m treated diabetic rats; blue filled triangle: TA3m treated diabetic rats; red open diamond: STZ-diabetic rats; black filled circle: normal control rats. As the hyperglycemic correction due to the first TA1m treatment diminished and fasting blood glucose levels started to rise, the experimental rats were injected a second time on the day 26^th^ with TA1m at 0.8 µg/gm body weight. The second treatment re-established fasting euglycemia.

The advantage of minicircle DNA in producing insulin efficiently in liver cells was underscored in a side-by-side comparison of TA1m with TA1 cloned in a conventional plasmid pENTR. One group of diabetic rats was treated with TA1m and the other group with an equimolar dose of TA1/pENTR. Diabetic rats treated with TA1m became normoglycemic within 1–2 days after treatment, whereas the TA1/pENTR treated rats remained hyperglycemic. Human insulin levels in sera of TA1m treated STZ-rats were ∼17-fold higher than TA1/pENTR treatment (22.1 mU/L versus 1.3±0.2 mU/L). Expression of human insulin mRNA in gene treated diabetic rats was readily detected only in livers, all the other tested organs showed an absence of insulin mRNA. This finding is consistent with the design of insulin expression constructs and our previously reported results [6].

### Insulin Production in Hepatocytes, *in vivo*

In vivo insulin production in livers of TA1m treated rats was readily evident in insulin stained liver sections (Fig. 9) and via mRNA levels in liver tissue (data not shown). The entire liver contained nearly randomly distributed insulin positive cells, as shown in a low magnification photomicrograph of a liver section from a TA1m treated diabetic rat (Fig. 9, panel A). We estimated that the hydrodynamic minicircle DNA delivery causes insulin expression in approximately 4–6% of liver cells. Hemotoxylin and eosin stained sections of livers from diabetic rats, harvested after 4 days and up to a month after TA1m treatment did not show any indication of T-cell infiltration (Fig. 9, panel C). We were also unable to detect any anti-insulin antibody in the serum of TA1m treated rats (data not shown). We were unable to detect insulin in sections of pancreata from STZ treated diabetic control as well as insulin gene treated rats (Fig. 9, panel E) when insulin in sections of pancreata from normal rats was readily detectable (Fig. 9, panel F).

**Figure 9:**
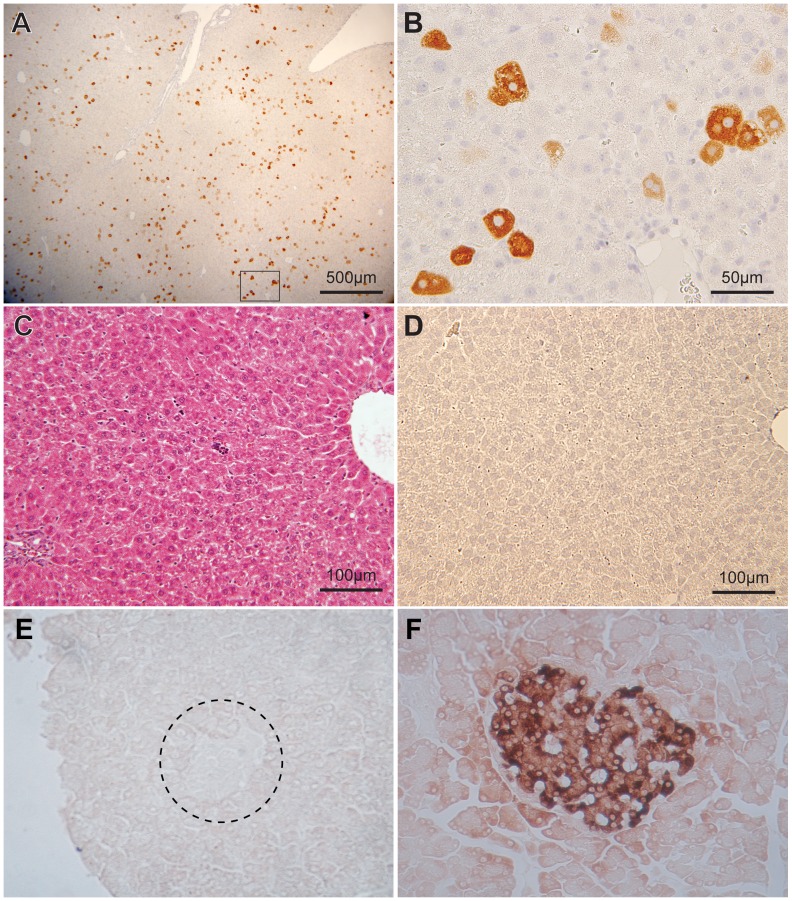
Detection of human insulin in liver sections of TA1m treated diabetic rats. Immunocytochemical detection of insulin in sections of livers from diabetic rats treated with TA1m was performed as described in methods. The scale bar in the low magnification view in the panel A corresponds to 500 µm. The panel B represents a higher magnification view of marked area in panel A. Individual insulin positive hepatocytes were clearly visible with a cytosolic stain (panel B); the scale bar represents 50 µm. The panel C shows an intermediate magnification view of hemotoxylin and eosin stained TA1m treated liver section; no overt T-cell infiltration was evident. The scale bar in panel C represents 200 µm. The panel D shows an intermediate magnification view of a section of liver from an untreated diabetic rat; no insulin staining was present. The scale bar in panel D corresponds to 200 µm. Sections of pancreata from each group of test and control animals were also subjected to immunocytochemical detection of insulin. Insulin positive cells were not detected in pancreata of STZ-treated diabetic rats before or after TA1m treatment; Fig. 9 E shows a representative section of pancreata from STZ-diabetic animal treated with TA1m, where an insulin negative islet can be seen (broadly outlined by dashed circle). Insulin in β-cells of normal rat pancreata was readily detectable (Fig. 9 F).

### Intraperitoneal Glucose Tolerance Test

The intraperitoneal glucose tolerance test employed a substantial glucose load (4gm/kg body-weight) to amply challenge the ability of experimental animals to dispose of glucose. Results showed that the serum glucose level in diabetic rats treated with TA1m peaked 15min after the intraperitoneal glucose injection and returned to normal in 30–45min (Fig. 10A). This pattern is similar to normal rats (Fig. 10B), where rat insulin levels peaked at 4.3±0.1 mU/L and returned to 0.44±0.27 mU/L at 2 hr. The human insulin levels in rat serum increased along with rise in glucose levels with a delay of only about 15min and rapidly declined to basal level (Fig. 10A) in a manner analogous to normal animals. In our previous studies using adenovirus vector, peak levels of human insulin in diabetic rats under similar conditions were approximately 10 fold lower, which paralleled suboptimal correction of ad libitum fed blood glucose levels.

**Figure 10:**
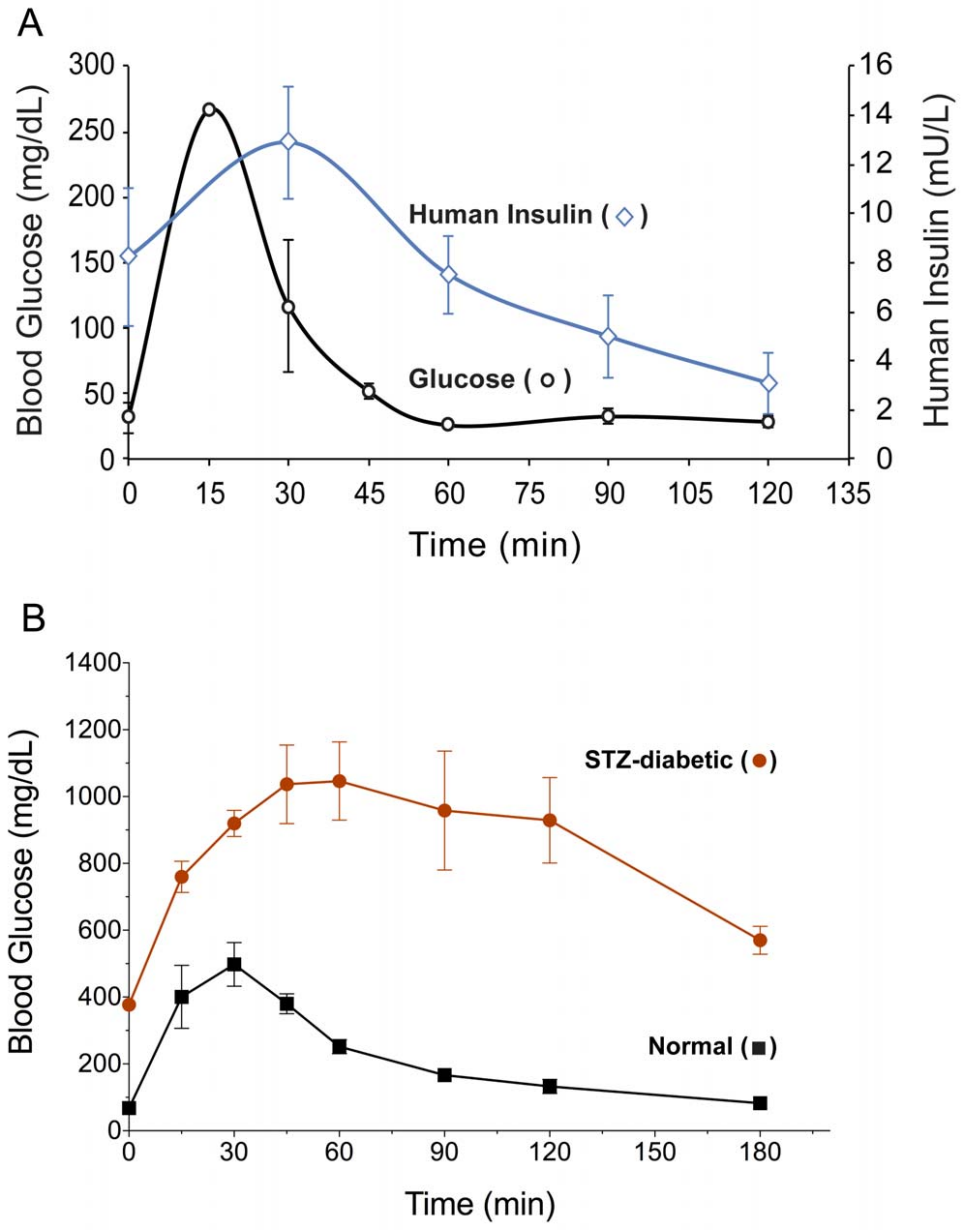
Glucose tolerance test. A group of young diabetic rats was treated with 0.8 µg/gm TA1m by high pressure tail vein injection, as described in methods. Control groups included age-matched STZ-diabetic and normal rats. Four days after TA1m treatment, all rats were fasted and intraperitoneally injected with 4gm glucose/kg body weight. Blood samples over a 2 hr period at the indicated time intervals were collected from each experimental rat, blood glucose and plasma levels of human insulin were measured. Fig. 10A: open black circle, blood glucose levels (mg/dl); open blue diamond, plasma insulin levels (mU/L). Blood glucose levels in normal control rats (black filled square) and STZ-diabetic rats (red filled circle) subjected to intraperitoneal glucose tolerance test are shown in Fig. 10B.

### Effect of TA1m Gene Therapy on Serum Lipid Levels and Liver Function

Uncontrolled or poorly regulated hyperglycemia in diabetes causes serious metabolic dysregulation, including hyperlipidemia, hypercholesterolemia, ketosis, impaired liver function, liver damage, weight loss, and development of cataracts. Our results show that a single TA1m treatment not only substantially corrects hyperglycemia, it also restores various diabetes-associated markers of metabolic dysregulation to normal levels (Table 1). Furthermore, the liver function is also restored to normal (Table 1).

**Table 1:** Effect of TA1m treatment on markers of liver damage and liver function.

Animal Groups	Triglycerides(mg/dl)	Cholesterol(mg/dl)	Aspartate Transaminase (U/L)	Alanine Aminotransferase (U/L)	Alkaline Phosphatase (U/L)	Plasma Albumin (g/dl)
TA1m treated	53±34	141±33	302±33	77±40	210±95	3.4±0.2
STZ-Diabetic	704±313	191±36	617±349	152±75	423±73	2.5±0.2
Normal Control	100±14	129±6	504±100	106±16	172±55	3.4±0.1

*Effect of TA1m treatment on markers of liver damage and liver function:* Two groups of rats (5 animals/group) were rendered diabetic by STZ. One group was injected with TA1m at 0.8 µg/gm body-weight via tail vein at high pressure. The second group of STZ-treated rats was used as an untreated diabetic control. A third group of normal rats was included as a control. After 10 days of TA1m treatment, blood samples were drawn for measurements of liver damage and liver function markers. The values shown in the table are Mean±Standard Deviation.

## Discussion

The search for alternative sources of glucose-regulated *in vivo* insulin production to treat T1DM is fueled by an increasing number of new onset T1DM patients in addition to a large preexisting pool of patients. This search is also driven by a severe shortage of donor pancreata, a situation that is unlikely to change soon, as well as less than optimal control of blood glucose level afforded by the contemporary therapies that are based on the use of exogenous insulin. In our studies we have chosen to express insulin in liver cells. Hepatocytes have a robust protein synthesis and constitutive protein secretory machinery, as well as mechanisms to sense and metabolically respond to changes in ambient glucose levels. To compensate for the absence of specific enzymes required for functional maturation of insulin in liver, the native cleavage sites have been modified. We previously showed that a combination of 3× GIREs and albumin promoter with modified insulin yielded glucose-dependent human insulin production [6]. However, the amount of insulin was insufficient to correct postprandial hyperglycemia. In the present study, we have dramatically increased the insulin output from our insulin constructs by adding various combinations of elements that improve the efficiency of transcription, mRNA processing, and insulin mRNA translation. As a consequence, not only can we correct fasting hyperglycemia in STZ-diabetic rats, we can also fully correct hyperglycemia in rats fed *ad libitum.*

Selection of a safe and effective vector for clinical gene therapy is crucially important. Because adenovirus vector is very efficient in transducing many types of cells, including hepatocytes, it has proven useful in testing newly developed insulin constructs in our studies. However, adenovirus is immunogenic and it can only be used once for a transient expression, making it unsuitable for long-term repeated treatments. Other viral vectors, such as recombinant adeno-associated virus (rAAV) and lentivirus that cause gene expression without chromosomal integration, have practical limitations due to their lower titers, and being less effective in transducing many types of cells, although various pseudo-types of rAAV can be prepared that target specific cells somewhat preferentially [40]. While the antigenicity of some of these viral vectors is reduced, it is not abolished [41]. Viral vectors that integrate into chromosomes can offer the benefit of long-term expression but they also carry the highest risk of being oncogenic. Some plasmid vectors used for gene therapy, such as treatment of hemophilia, have been reported to be therapeutically effective [42]. It should be noted that in those cases the gene products had a relatively long half-life, which is unlike insulin in our experiments which has a short circulating life-span and must be synthesized in a regulated manner in substantial quantities. In most cases of plasmid use, duration of gene expression tends to be short. Various investigators have identified many aspects of conventional plasmids that contribute to this limitation. Among the contemporary viral and non-viral vectors, minicircle DNA appears to have many desirable traits, and was therefore chosen in our study. Minicircle DNA is free of extraneous bacterial or viral DNA and it does not integrate into chromosomes. Lacking an antibiotic resistance gene commonly found in conventional plasmids, it poses no danger of incorporation into endogenous bacteria either directly during treatment or alternatively through a lateral movement from liver cells [43]. A virtual absence of bacterial and viral sequences improves the likelihood that minicircle DNA will not become the target of the immune system or gene silencing. Indeed, minicircle DNA has been reported to be effective in long-term expression of genes [44].

We observed that minicircle DNA TA1m is able to correct hyperglycemia in diabetic rats more effectively than the highly infective adenovirus vector (Ad-TA1). At the highest tested dose (1.0 µg/gm) TA1m treatment caused a mild hypoglycemia in diabetic rats, fed *ad libitum* (mean glucose d2–9 post treatment: 72 mg/dl). The reduction in hyperglycemia was DNA dose dependent (mean blood glucose, d2–9 post treatment: 113 mg/dl at 0.75 µg/gm, 200 mg/dl at 0.50 µg/gm, 319 mg/dl at 0.25 µg/gm) (Fig. 5A, B). Unlike determination of viral titers, which tends to be inaccurate, precise minicircle DNA concentration measurements by simple methods will allow for better dose-response studies. We expect that an optimized dose of DNA would eliminate or greatly reduce the potentially dangerous hypoglycemia. A full restoration of rate of weight gain in young diabetic rats (46.3±5.4gm) was noted at 0.5–1.0 µg/gm minicircle DNA doses. Thus, groups of diabetic rats treated with 0.5 µg/gm, 0.75 µg/gm, and 1.0 µg/gm TA1m DNA gained weight at a rate of 6.7±2.3gm/day, 7.1±2.5gm/day, and 6.5±1.8gm/day, respectively; age-matched normal control rats gained weight at the rate of 7.2±1.7gm/day. In contrasts, diabetic rats treated with the lowest TA1m DNA dose (0.25 µg/gm) gained weight at the lowest rate (4.8±3.3gm/day) but even at this suboptimum correction of hyperglycemia at low dose TA1m treatment, the difference in weight gain rate compared to untreated age-matched diabetic controls (2.4±2.2gm/day) was statistically significant (Fig. 6). Furthermore, our preliminary results also indicate that when the effect of the first treatment begins to diminish, a second treatment can restore glucose control (Fig. 8), which is not possible when immunogenic viral vectors are used for the first treatment.

The advantage of using minicircle DNA to efficiently produce insulin in liver cells was evident when an equal gene copy dose of TA1m and TA1 cloned in a conventional plasmid pENTR, was compared by treating matched groups of diabetic rats. The plasmid pENTR contains a conventional center for replication in bacteria and the kanamycin resistance gene. Diabetic rats treated with TA1m became normoglycemic within 1–2 days after treatment, whereas the TA1/pENTR treated rats remained hyperglycemic. Rat serum levels of human insulin, which could only be produced by our insulin constructs, were ∼17-fold higher in rats treated with TA1m as compared to the TA1/pENTR treatment.

A robust insulin production in livers of TA1m treated rats was observed in insulin stained liver sections (Fig. 9). We roughly estimated that the hydrodynamic minicircle DNA delivery causes insulin expression in approximately 4–6% of liver cells. High pressure tail vein injection of plasmid solution is reported to cause gene expression in 10% or more liver cells [38], [45]. It must be emphasized that hepatocytes cannot store insulin; all of it is constitutively secreted. It is possible that in some hepatocytes making less insulin, while concurrently secreting it, insulin levels may fall below the limits of immunocytochemical detection, and consequently appear negative. Thus, the true number of hepatocytes producing insulin may be somewhat higher than observed. Hemotoxylin and eosin stained sections of livers from diabetic rats, harvested after 4 days and up to a month after TA1m treatment did not show any indication of T-cell infiltration or the presence of anti-insulin antibody in the serum of TA1m treated rats. Our results are consistent with previously published studies reporting that insulin expression in liver does not cause the hepatocyte to become a target of immune attack [46].

The results of intraperitoneal glucose tolerance tests using a high glucose load (4gm/kg) in fasting rats treated with TA1m were striking; the glucose level rose above 110 mg/dl for only about 30 min and fully corrected to the starting level in <45 min. It is interesting to note that at 60 min post-bolus glucose exposure, when the blood glucose level was already normalized, a moderate amount of human insulin was present in plasma (7.5±1.6 mU/L) which continued to decrease while the blood glucose remained normal. By two hours post-glucose, insulin levels had declined to 3.0±1.3 mU/L. The excursion of blood glucose level following the high glucose load was substantial. But this result is unsurprising due to the nature of this treatment, which relies on glucose-induced *de novo* synthesis of insulin. In contrast, β-cells in normal animals respond to hyperglycemia by near instantaneous secretion of stored insulin. Remarkably, the duration of elevated glucose in TA1m treated rats was relatively short due to a rapid and vigorous human insulin production response as evidenced by insulin peak in the sera of rats, lagging only ∼15 min behind the glucose peak levels (Fig. 10). Likewise, a dynamic attenuation of glucose-induced production of insulin as the glucose level is normalized, was also expected to show some delay. We expected that *in vivo* counter-regulatory mechanisms would adequately control potential transient hypoglycemia and the data confirmed our expectations. Since we employed intraperitoneal injections as the method for glucose loading, insulinotropic effects of gut hormones (incretins) are less likely to be relevant. Furthermore, use of streptozotocin can disrupt several intestinal hormones, including glucagon like peptide 1 (GLP1). Reduced or lack of insulinotropic activity of incretins is likely to add to the delay in observed insulin function. From the perspective of therapy, such excursions could be safely managed by avoiding very large meals. We also noted that a single TA1m treatment effectively protected diabetic rats from many of the common long-term diabetes-associated damages, as judged by their serum lipid profile, liver damage and liver function markers. With the unique construct design used in this study, insulin gene therapy provides glycemic control for approximately 1 month but metabolic benefits extend well beyond that period. The TA1m treated diabetic rats exhibited an absence of cataracts and a healthy general appearance for up to 2–3 months, even though they were no longer normoglycemic. It appears that even suboptimal insulin production is enough to counteract many drastic systemic damages. We suggest that the overall control of hyperglycemia in diabetics following the TA1m gene therapy combined with a sensible dietary practice would be superior to the current commonly used insulin injections, and would minimize long-term diabetes-associated systemic damages.

To our knowledge, this is the first study to show a minicircle DNA-based insulin gene therapy resulting in glucose-regulated insulin production from liver with a potential to treat T1DM. A safe and effective treatment for T1DM could be developed by combining TA1m DNA with new methods of *in vivo* gene delivery. Alternatively, liver tissue could be procured by a needle biopsy, hepatocytes or progenitor cells isolated, expanded, and transduced *ex vivo* with TA1m and transplanted into the recipient’s liver. In this context, it is interesting to note that very efficient methods for hepatocyte derivation from iPS cells are already available [47] which could allow the use of hepatocytes from autologous derivation in insulin gene-therapy. The *ex vivo* hepatocyte transduction and transplantation of insulin producing cells for T1DM treatment is more complex but has the advantage of making it possible to pretest insulin-producing cells to optimize the treatment, and minimize the systemic exposure of TA1m to the recipient. The liver-based treatment of T1DM by TA1m gene therapy avoids chromosomal integration related concerns and is unlikely to require immunosuppression.
